# Application of Electrolyzed Hydrogen Water for Management of Chronic Kidney Disease and Dialysis Treatment—Perspective View

**DOI:** 10.3390/antiox13010090

**Published:** 2024-01-11

**Authors:** Masaaki Nakayama, Shigeru Kabayama, Mariko Miyazaki

**Affiliations:** 1 Kidney Center, St. Luke’s International Hospital, Tokyo 104-8560, Japan; 2 Division of Blood Purification, Tohoku University Hospital, Sendai 980-8574, Japan; shigeru.kabayama.c1@tohoku.ac.jp (S.K.); mamiyaza@med.tohoku.ac.jp (M.M.); 3 Graduate School of Science, Technology & Innovation, Kobe University, Kobe 657-8501, Japan; 4 Nihon Trim Co., Ltd., Osaka 530-0001, Japan; 5 Division of Nephrology, Rheumatology and Endocrinology, Graduate School of Medicine, Tohoku University, Sendai 980-8574, Japan

**Keywords:** electrolyzed hydrogen water, antioxidant, chronic kidney disease, hemodialysis

## Abstract

Chronic kidney disease (CKD), which is globally on the rise, has become an urgent challenge from the perspective of public health, given its risk factors such as end-stage renal failure, cardiovascular diseases, and infections. The pathophysiology of CKD, including dialysis patients, is deeply associated with enhanced oxidative stress in both the kidneys and the entire body. Therefore, the introduction of a safe and widely applicable antioxidant therapy is expected as a measure against CKD. Electrolyzed hydrogen water (EHW) generated through the electrolysis of water has been confirmed to possess chemical antioxidant capabilities. In Japan, devices producing this water have become popular for household drinking water. In CKD model experiments conducted to date, drinking EHW has been shown to suppress the progression of kidney damage related to hypertension. Furthermore, clinical studies have reported that systemic oxidative stress in patients undergoing dialysis treatment using EHW is suppressed, leading to a reduction in the incidence of cardiovascular complications. In the future, considering EHW as one of the comprehensive measures against CKD holds significant importance. The medical utility of EHW is believed to be substantial, and further investigation is warranted.

## 1. Introduction

### 1.1. Historical Background of Electrolyzed Hydrogen Water

“Electrolyzed hydrogen water” (EHW) refers to the water generated on the cathode side during the electrolysis of water. Chemically, it is characterized as weakly alkaline (with a pH of 9.0 or higher but lower than 10.0) and contains hydrogen molecules (H_2_). The concentration of H_2_ varies depending on the model of the generator, but it can be adjusted arbitrarily to 100–1300 ppb immediately after generation by adjusting the electrolysis intensity and water flow rate. The generator of this water is called an “electrolyzed water generator”, also known as a water ionizer. In Japan, it received medical device approval from the former Ministry of Health and Welfare in 1945 as a home drinking water generator. Furthermore, it has undergone double-blind trials, confirming its effectiveness in improving gastrointestinal symptoms such as abdominal discomfort, fullness, diarrhea, and constipation, i.e., significant global improvement of abdominal symptoms in the group with EHW as compared with the control [1]. Currently, approximately 200,000 units of this device are manufactured and sold annually in Japan, and the consumption of EHW is assumed to be incorporated into daily life at a certain level [2].

### 1.2. Cross over with Hydrogen Medicine

In the scientific exploration of EHW, Shirahata et al. first demonstrated its antioxidant capability in 1997 [3]. The chemical characteristics of this water include its ability to suppress the generation of superoxide anions and promote the decomposition of hydrogen peroxide. It has been shown to inhibit the production of reactive oxygen species and exhibits catalase-like activity and biological effects such as suppression of apoptosis via oxidative stress [4] and the extension of the lifespan of nematodes through its antioxidant action [5]. Regarding the mechanism of this antioxidative effect, Shirahata and others suggested that the mechanism of this antioxidative effect involves factors such as the influence of nano-sized platinum particles released from the electrodes used in electrolysis [5,6] as well as changes in water molecules due to the electrolysis of water. However, many details of the precise mechanism remain unclear.

In 2007, Ohsawa et al. reported that inhalation of H_2_ suppresses the expansion of cerebral infarcts caused by brain artery clamping, suggesting the involvement of direct hydroxyl radical elimination by H_2_ [7]. This triggered the development of hydrogen medicine, and various organ-protective effects from the antioxidant and anti-inflammatory effects of H_2_ have been confirmed in animal experiments [8]. In this context, the mechanism of the biological effects of EHW is now assumed to involve H_2_ [9].

Currently, research on the application of H_2_ for human disease prevention and treatment is underway [10]. In this sense, the situation in Japan, where electrolyzed hydrogen water generators are already in use among the general public, is intriguing. When considering real-world medical applications, we believe that the potential medical significance of EHW in preventing disease onset and suppressing exacerbation in the pre-symptomatic state, as well as in preventive healthcare, is significant. Cross-sectional comparative studies targeting healthy individuals have been conducted [11], reporting significantly lower oxidative stress values in the blood of daily EHW consumers compared to non-consumers, as well as significantly lower levels of blood urea nitrogen, a kidney function indicators.

This paper summarizes the latest findings on the biological effects of H_2_ and discusses the medical significance of applying EHW to chronic kidney disease (CKD) and dialysis therapy, which are critical concerns to public health.

## 2. Latest Insights into H_2_ Biology Research—Brief Summary

H_2_ has been demonstrated to possess anti-inflammatory, antioxidant, and anti-endoplasmic reticulum (ER) stress properties, and its involvement in the regulation of apoptosis, autophagy, and pyroptosis has been elucidated [12,13,14,15,16,17,18]. In this regard, H_2_ can be considered a unique molecule that influences fundamental biological responses. Although mechanisms for the multifaceted effects of H_2_ have been proposed, the fundamental processes still remain unclear. Ohsawa et al. suggested the possibility of H_2_ directly scavenging hydroxyl radicals [7]; however, subsequent studies revealed the effectiveness of H_2_ preconditioning in organ protection. As this phenomenon cannot be solely explained as due to chemical reactions of H_2_, it is speculated that H_2_ may impact the body’s inflammatory and antioxidant systems, activating the body’s defense mechanisms [11]. In this context, recent attention has been drawn to the relationship between H_2_, the redox system in the body, and the mitochondria [14,15,16].

**Figure 1:**
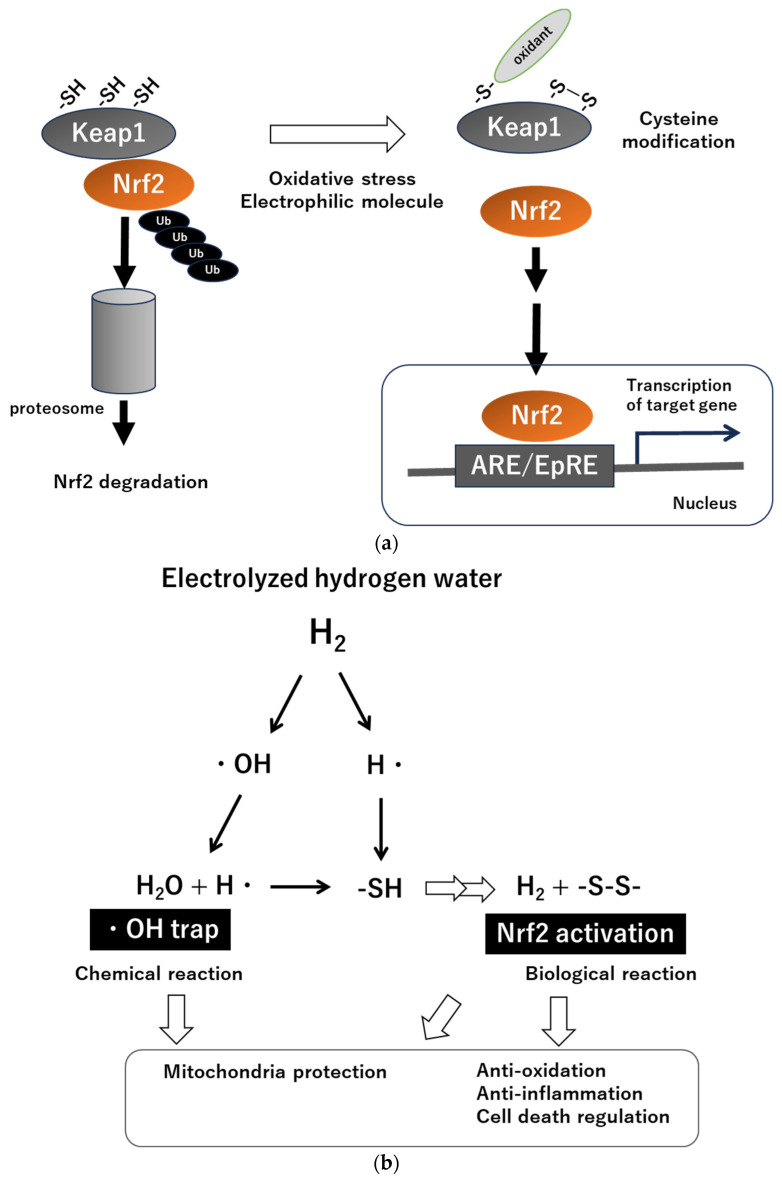
(**a**) Stress-sensing mechanisms of the Keap1-Nrf2 system. Abbreviations: Nrf2, nuclear factor–erythroid 2-related factor 2; Keap1, Kelch-like ECH-associated protein 1; Ub, ubiquitin; -SH, thiol; ARE, antioxidant response element; EpRE, electrophile responsive element. (**b**) Mechanisms underlying the manifestation of biological effects of electrolyzed hydrogen water (hypothesis). Abbreviations: H_2_O, water; OH, hydroxyl radical; H, hydrogen radical; -SH, thiol; -S-S-, disulfide.

Nuclear factor–erythroid 2-related factor 2 (Nrf2) and Kelch-like ECH-associated protein 1 (Keap1) serve as the master regulators of cellular redox in the body [19,20] (Figure 1a). Keap1 is present in the cytoplasm and functions as a stress sensor, serving as an enzyme that contributes to the degradation of Nrf2; i.e., in non-stressed cellular conditions, Nrf2 is sequestered by Keap1 and degraded by the ubiquitin–proteasome system. When cells are exposed to stimuli such as electrophilic substances, reactive oxygen species, or endoplasmic reticulum stress, Nrf2 is released from Keap1 inhibition and becomes activated as transcription factor to induce expression of antioxidant response element (ARE)/electrophile responsive element (EpRE) of genes, which include over 200 genes, including major antioxidant and anti-inflammatory molecules.

Accumulated reports from animal experiments indicate that H_2_ administration enhances Nrf2 expression [21,22,23,24,25,26,27]. However, Nrf2 expression is supposed to be triggered by oxidative stress stimuli. From this perspective, there is a possibility that H_2_ induces a so-called hormesis phenomenon [28]. Indeed, the potential of H_2_ to induce mild oxidative stress has been reported [28,29,30], and activation of antioxidant systems via oxidative stimulation cannot be denied. This is analogous to the body’s response to exercise [31]. We will outline our hypothesis regarding the mechanism of H_2_ to activate Nrf2 (Figure 1b). The core of the matter lies in the fact demonstrated by Ohsawa et al. [7], who reported that H_2_ directly scavenges hydroxyl radicals in the form of electrophilic hydrogen radicals (H:H → H + H, H + OH → H_2_O). However, in general, a single electrophile could have both protective and toxic effects on cells. It is known that electrophiles react with nucleophiles, including protein thiols (-SH), such as those found in reduced glutathione (GSH) or guanine bases in DNA [32]. Each of them has an unshared pair of electrons, and the reaction of an electrophile with the -SH of a cysteine residue results in alkylation [33], leading to the decrease of the reductive capacity of cells, i.e., depletion of GSH. If we consider this phenomenon in terms of hydrogen radicals, theoretically it is possible that hydrogen radicals could react with GSH, resulting in a reduced reductive capacity of cells. Hydrogen radicals may also react with the -SH of cysteine residues in Keap1, leading to the generation of H_2_ and modification of the cysteine residue of Keap1 (disulfide reaction, -S-S-), which may trigger the activation of Nrf2 action. However, the responses of electrophiles are generally characterized by dose-response [34]; therefore, different doses of H_2_ and accompanying levels of hydrogen radicals may induce different responses. At present, the fate of hydrogen radicals in cells is completely unknown and requires further investigation.

Mitochondria, the energy production mechanism, are the major source of reactive oxygen species in cells. It is assumed that the small molecule H_2_ is easily distributed within cells, and therefore, it is expected to be directly involved with mitochondria. It has been demonstrated that H_2_ supplementation is related to the preservation and maintenance of mitochondria [35,36]. The proposed mechanism suggests that H_2_ captures excess reactive oxygen species in mitochondria, preserving them from oxidative stress damag, and ultimately exhibiting organ protection effects [14,15,16]. Recently, a connection between the gut microbiota and mitochondrial function has been suggested [37,38,39,40]. H_2_ is involved in preserving mitochondrial function, while the gut microbiota serves as a source of H_2_ production in the body [41]. Future investigations are expected to explore whether H_2_ acts as a missing link between the gut microbiota and mitochondrial function.

In recent reports, it has been revealed that the consumption of hydrogen-rich water can have an impact on the intestinal microbiota [42]. In this context, the intriguing point is whether externally adding H_2_ may enhance the interconnection between the gut microbiota and mitochondria within the body, potentially amplifying the anti-stress effects in the living organism.

In summary, recent findings have been summarized, but many aspects of the fundamental mechanisms and starting points of H_2_’s actions on cells and the body remain unknown. However, considering that no adverse effects of H_2_ on the body have been confirmed, the clinical application of H_2_ has become a realistic challenge. Within this context, establishing methods of H_2_ administration that can demonstrate the effectiveness of H_2_, taking into account the characteristics of the disease, is considered a challenge.

## 3. Oxidative Stress in CKD and Dialysis Therapy

### 3.1. Oxidative Stress and Inflammation as Fundamental Pathophysiology of CKD (Figure 2)

CKD is a state of decreased glomerular filtration rate or high albuminuria. The prevalence of CKD being a risk factor for cardiovascular complications and progression to end-stage renal failure is increasing globally, driven by the spread of lifestyle diseases and aging, accounting for 15–20% of adults globally [43]. CKD presents an urgent challenge to public health. The major contributors to the onset and exacerbation of CKD are lifestyle diseases such as hypertension, diabetes, and obesity. Therefore, comprehensive lifestyle management, including blood pressure control, obesity correction, and exercise habits, is crucial in CKD prevention. On the other hand, one clinically significant factor affecting CKD progression is the occurrence of episodes of acute kidney injury (AKI) during the course of the disease [44]. The central pathology of this condition is renal ischemia. Even after the patient recovers from AKI, the subsequent progression of CKD is accelerated. Hence, avoiding factors that induce or worsen renal ischemia, such as drugs, infections, and dehydration, or implementing renal protective measures during ischemia, becomes crucial.

**Figure 2:**
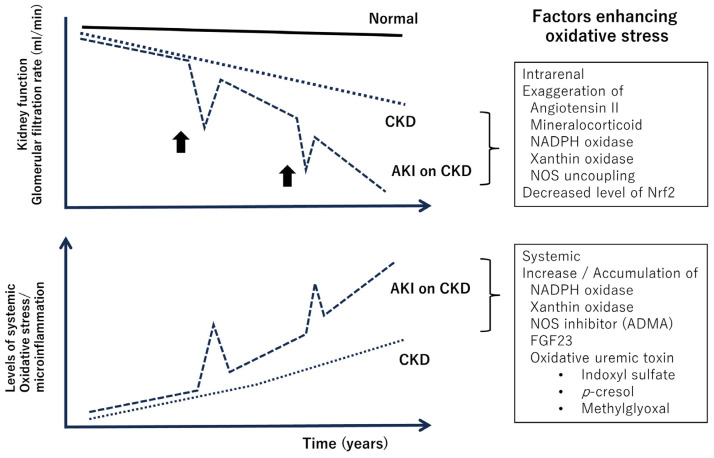
Decline of kidney function (above) and systemic enhancement of oxidative stress and microinflammation during the clinical course of chronic kidney disease (below). Oxidative stress within the kidneys promotes renal damage [45,46]. Bold arrows indicate episodes of ischemia, inflammation in the kidney, and exposure to nephrotoxic agents. As renal function declines, systemic oxidative stress increases [47]. Abbreviations: CKD, chronic kidney disease; AKI, acute kidney injury; NOS, nitric oxide synthase; Nrf2, nuclear factor–erythroid 2-related factor 2; ADMA, asymmetric dimethylarginine; FGF23, fibroblast growth factor 23.

The progression of CKD is fundamentally associated with inflammation and fibrosis in renal interstitial tissues, and oxidative stress is believed to play a substantial role in this process [45,46]. For example, in CKD, the expression of Nrf2 in the kidneys is decreased, leading to increased oxidative stress and involvement in inflammation within the kidneys [48]. On the other hand, oxidative stress has been implicated in the pathophysiology of kidney damage in hypertension model rats, and antioxidants have been confirmed to be effective in suppressing kidney damage [49,50]. Given these facts and the extremely high prevalence of hypertension among CKD patients [51], oxidative stress is believed to play a central role in CKD progression.

In real-world, clinically available medications such as angiotensin receptor blockers (RAS inhibitors), which inhibit the activation of NADPH oxidase with angiotensin II, are assumed to be involved in their reno-protective mechanism [52]. Regarding the suppressive effect of SGLT2 inhibitor on CKD progression, one of the mechanisms is the suppression of enhanced glycolysis in the proximal tubule cells, which generate oxidative substances such as methylglyoxal [53]. Furthermore, the clinical significance of oxidative stress in kidney damage is suggested by the improvement of renal function in diabetic kidney disease with bardoxolone methyl, an Nrf2 activator [54].

On the other hand, a characteristic feature of enhanced oxidative stress in CKD is that it is not only localized in the kidneys; systemic enhancement has also been identified, which is assumed to be related to an increased risk of cardiovascular disease and infection [47]. Indoxyl sulfate, p-cresol, and methylglyoxal, which accumulate in the body due to decreased kidney function, are oxidative uremic toxins. Indoxyl sulfate enhances NF-kB expression and inhibits Nrf2 expression [55], p-cresol increases NADPH oxidase expression [56], and methylglyoxal enhances oxidative stress through chemical reactions with H_2_O_2_ [57]. Phosphate levels increase in the blood due to decreased kidney function, and this is associated with increased FGF23 and decreased klotho, both of which are related to increased oxidative stress [58].

### 3.2. Complications of Hemodialysis Therapy and Oxidative Stress/Inflammation (Figure 3)

Patients undergoing hemodialysis have a poorer life prognosis compared to the general population due to complications such as cardiovascular disease and infections. It has been noted that oxidative stress and microinflammation in the body are elevated in dialysis patients, and this degree of elevation is closely related to life prognosis [47]. The causes of increased oxidative stress in hemodialysis patients include factors related to patients’ medical background, including uremic state existing since the pre-dialysis period; hemodialysis-related oxidative factors, such as the presence of endotoxins in dialysis fluid; and biocompatibility of dialysis equipment and materials [59]. Regarding biocompatibility, although various types of dialyzers have been developed, challenges still exist. White blood cell stimulation and complement activation occur upon contact between dialysis equipment and blood, and with these, myeloperoxidase (MPO) released from neutrophils can produce powerful prooxidative substances, such as hypochlorous acid in the presence of hydrogen peroxide. Therefore, bio-incompatibility during dialysis can serve as a trigger for exacerbating biologically oxidative stress and inflammation. Indeed, MPO levels in blood increase with hemodialysis, and MPO is a significant risk factor for patient prognosis [60]. The mechanisms of MPO release from neutrophils during dialysis could include physiological degranulation, passive cell rupture, and active reactions, such as apoptosis-induced necrosis, and NETosis. However, there are not sufficient data to determine which mechanism is significant. At present, there are insufficient data on dialysis equipment and systems that can suppress an increase of MPO.

**Figure 3:**
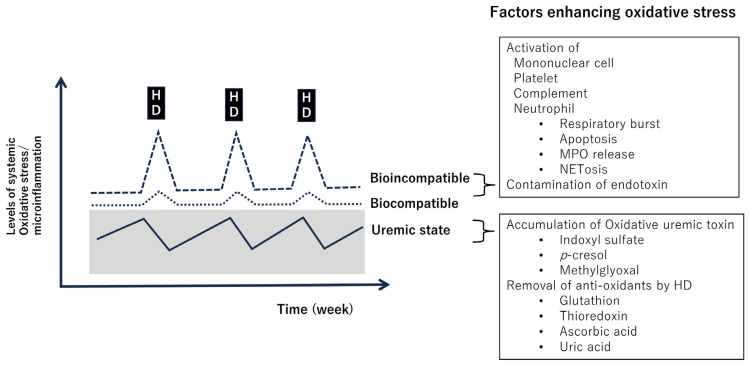
Enhancement of oxidative stress and microinflammation in patients on hemodialysis. Dialysis removes antioxidant substances [47]. On the other hand, the bio-incompatible dialysis membrane stimulates monocytes, platelets, complement, and neutrophils [59]. Abbreviations: HD, hemodialysis; MPO, myeloperoxidase.

## 4. H2 Intervention for CKD and Hemodialysis

Given the multifaceted involvement of oxidative stress in various pathologies, antioxidant therapy is considered extremely significant. However, the expected results are not always obtained in interventions employing antioxidants, including for CKD [61]. Reactive oxygen species are a double-edged sword, having both detrimental effects on the body and being crucial for the body’s defense. In this sense, excessive oxidative stress should be suppressed, but the degree of suppression should be at a level that does not compromise the benefits of reactive oxygen species to the body [15]. To date, while many preclinical studies using H_2_ have confirmed organ-protective effects and correction of metabolic abnormalities through its antioxidant and anti-inflammatory effects [12,13,14,15,16,17,18], no severe side effects of H_2_ loading have been observed. Therefore, the clinical application of antioxidant therapy with H_2_ is considered a realistic challenge. The following summarizes the research on CKD and dialysis-related topics.

### 4.1. Pre-Clinical Studies of H_2_ in CKD Models

The reno-protective effects of H_2_ have been reported in various models of kidney diseases [62,63] via H_2_ administration through drinking water, intraperitoneal administration, and inhalation. These studies involve acute models such as acute kidney injury via ischemia–reperfusion of renal artery clamp [64,65,66,67,68,69,70,71,72,73,74], allograft rejection [75,76], drug-induced nephrotoxicity [77,78], renal calculi [79,80], and renal fibrosis via ureteral ligation [81,82,83]. However, reports on CKD are limited [21,84,85] (Table 1).

In a study of spontaneously hypertensive rats, the effects of drinking hydrogen water (1.2 mg/L) freely for 3 months under salt reduction were investigated [84]. The results showed no difference in the onset of high blood pressure compared to the control group. However, histologically, kidney damage was suppressed, and a significant increase in antioxidant substances and a significant decrease in proinflammatory substances were confirmed in rats on hydrogen water compared to the control group. Although hydrogen water drinking did not suppress the onset of hypertension itself, it was suggested that H_2_ could inhibit the process of oxidative kidney damage associated with hypertension.

In Dahl salt-sensitive rats, which exhibit high blood pressure, kidney damage, and cardiac hypertrophy in response to salt loading [21], the effects of drinking EHW (0.4 mg/L) freely for 12 months under salt reduction were investigated. The results showed delayed onset of high blood pressure, and histologically, kidney damage and myocardial hypertrophy were suppressed compared to the control group. In the hydrogen water-drinking group, Nrf2 expression in cardiac tissues was enhanced, suggesting increased resistance to oxidative stress in the body.

It is known that episodes of renal ischemia in CKD accelerate the progression of subsequent kidney damage. This pathophysiology is thought to involve not only localized renal ischemia but also the induction of inflammation in non-ischemic areas initiated by localized renal ischemia [86]. In experiments using Dahl salt-sensitive rats raised drinking EHW freely, the effects of contralateral kidney effects following unilateral renal ischemia were investigated [85]. The degree of renal tissue damage in the EHW drinking group was significantly lower than that in the control group, and oxidative stress in the same site was suppressed compared to the control group.

Summarizing these considerations, free drinking of hydrogen-containing water suggests a suppression of renal tissue damage in a model of hypertensive rats. The mechanism is presumed to be related to the alleviation of renal stress due to microcirculatory changes in the kidneys, which are mediated through systemic hypertension, as hydrogen-containing water did not directly suppress the onset of hypertension itself [21,84]. On the other hand, inhalation of H_2_ has been reported to ameliorate hypertension, implying involvement of changes in the balance of the sympathetic and parasympathetic nervous systems [87]. Considering the reported ability of parasympathetic nerve stimulation to suppress acute renal injury [88], it may be possible to hypothesize an influence of H_2_ on the autonomic nervous system balance in the mechanism of inhibiting renal injury in CKD rats.

### 4.2. Clinical Studies of H_2_ Intervention in Related to CKD Pathologies

Up to now, no intervention studies with H_2_ have been reported in CKD patients. However, regarding CKD risk factors, such as diabetes mellitus (DM), metabolic syndrome, and hypertension, six clinical trials were reported to explore the potentials of H_2_ [89]. Among them, three studies may indicate the potential reno-protective effect, i.e., decreased oxidative stress marker and/or increased antioxidants in urine, by drinking hydrogen-rich water. Kajiyama et al. [90] conducted a double-blind cross-over trial of drinking 900 mL/d of hydrogen-rich pure water (1.2 mg/L) for 8 weeks in 30 patients with type 2 DM and 6 with impaired glucose intolerance. Intake of hydrogen-rich water was associated with significant decreases in urinary 8-isoprostanes, and there was a trend of decreased serum oxidized LDL and increased plasma levels of extracellular superoxide dismutase, which may indicate the amelioration of decreased oxidative stress in the body including the kidneys. Ogawa et al. [91] conducted a double-blind trial in which type 2 diabetes patients were given EHW for free consumption (1.5~2.0 L/day) for three months (23 patients on EHW, 20 on filtered water), and they found significant improvements in insulin resistance in those with high insulin resistance, and an amelioration of enhanced serum d-ROM, an oxidative stress marker, in the EHW group. In a secondary analysis of this study, drinking EHW significantly increased eGFR at 3 months as compared to the basal level, and significantly decreased the change in urinary 8-OHdG excretion (ng/mgCr), an oxidative stress marker (oral presentation by Ogawa et al. at the 62nd annual meeting of the Japanese Society of Nephrology, Nagoya, 2019). Nakao et al. [92] performed an open-label one-arm pilot study of drinking HRW (~1 mmol H_2_/day) for 8 weeks in 20 subjects with metabolic syndrome, and they reported a significant increase of SOD in urine; a significant decrease of TBARS, an oxidative stress marker in urine; and a significant decrease of serum creatinine levels, which indicate the reno-protective action of drinking HRW.

Regarding the impact of H_2_ on hypertension, Liu et al. [93] examined the effect of a mixture of H_2_–oxygen (O_2_) gas inhalation on middle-aged and elderly hypertensive patients for four hours daily over a two-week period (20 cases with the H_2_ + O_2_ gas, 29 with placebo air). As a result, the group inhaling the mixed gas showed a significant reduction in brachial systolic blood pressure and nighttime blood pressure measured via ambulatory blood pressure monitoring (ABPM). This effect was more pronounced in the elderly. Additionally, levels of angiotensin II and a certain aldosterone value were significantly decreased. The improvement of BP control accompanying decreased levels of plasma angiotensin II and aldosterone may well indicate the risk reduction for kidney damage.

Given that diabetes and hypertension are deeply involved in the progression of CKD, measures to correct these conditions are expected to contribute to the management of CKD. In clinical studies, the administration of hydrogen-containing water was within the scope of daily life without causing inconvenience, suggesting the potential for societal implementation. It is anticipated that future research will involve long-term investigations of a larger number of cases.

In addition, as mentioned in the introduction, the improvement in gastrointestinal symptoms (such as constipation) through the consumption of EHW has been confirmed [1,9]. Constipation is a risk factor for the presence of CKD [94], and it is hypothesized that this may be influenced by substances, such as indoxyl sulfate, produced within the intestinal tract [95]. It should be considered whether the improvement of constipation through the consumption of EHW affects the pathophysiology related to CKD progression, and this is an aspect that should be investigated in the future.

### 4.3. Clinical Studies Using EHW for Hemodialysis

The clinical application of hydrogen-rich water, specifically in hemodialysis, has been a focus of investigation. The initial report by Huang et al. demonstrated a reduction in inflammatory markers in hemodialysis using electrolyzed water [96,97]. Furthermore, it was reported that ERW enhances dissociation of indoxyl sulfate from albumin as an underlying mechanism [98]. However, widespread recognition of this treatment system did not occur. Subsequently, Nakayama et al. focused on the H_2_ concentration in electrolyzed dialysis fluid and proposed a treatment system based on this feature, leading to increased recognition of the system [99]. Currently, in Japan, it is estimated that over 30 facilities have introduced the hemodialysis system, with over 3000 treated patients. Table 2 summarizes these reports [30,100,101,102,103,104,105,106].

In hemodialysis, the dialysis solution required for a standard hemodialysis treatment exceeds 140 L per person per session. Dialysis solution itself has chemical oxidative stimuli (bio-incompatibility), but its character is suppressed in the solution created with EHW. As a result, in hemodialysis using EHW, a decrease in oxidative stress and inflammatory markers of patients has been confirmed. Furthermore, with long-term continuation of this treatment, the redox state of patients approaches that of healthy individuals [102].

Next, what does such a change in the internal environment bring clinically? One is the improvement in the overall prognosis of patients. In a prospective observational study comparing the outcomes of an EHW treatment group and a conventional dialysis group, a 41% significantly lower hazard ratio for the composite endpoint (total death, new stroke, new cardiovascular disease, lower limb amputation) was observed in the EHW group over a 5-year observation period [104]. Another potential improvement is in the most important patient-reported outcome, dialysis-related fatigue. Dialysis-related fatigue is both an inhibitory factor for quality of life and an independent risk factor for life prognosis. In EHW hemodialysis, the reduction of this dialysis-related fatigue has been reported in several observational studies [30,103,105,106]. The mechanism may involve the suppression of oxidative stress stimulation initiated by MPO induced by dialysis [30], changes in the balance of the sympathetic and parasympathetic nervous systems [105]. The H_2_ concentration in the dialysis solution influenced the patients’ reported outcome, i.e., more anti-fatigue effects at 150 ppb than an average of 50 ppb [106]. Future studies applying a novel device, which could simultaneously monitor H_2_ concentration in the dialysate [107], are expected to investigate the optimal H_2_ levels for the comprehensive patients’ outcome via randomized controlled study.

## 5. Questions and Challenges of EHW for CKD and Dialysis

### 5.1. Target Organs of H_2_

In animal experiments and clinical studies, promising results have been reported for the medical application of EHW. The concentration of H_2_ in drinking water in the study [91] ranged from 0.35 to 0.49 mg/L (350 to 490 ppb), and in dialysate, it ranged from 30 to 199 ppb [30,100,101,102,103,104,105,106]. The former involved effects through intestinal absorption, while the latter involved direct administration into the blood vessels. When the subjects drank EHW, a transient increase in exhaled H_2_ occurred relatively promptly (a few minutes later), suggesting that the absorption and diffusion of H_2_ into the body after drinking occurs rapidly, regardless of water absorption. In this case, the effect of H_2_ was expected to extend across multiple organs. In the latter case, H_2_ that diffused into the blood was transported directly to the heart and lungs, but at this point, most of the H_2_ was supposed to be excreted in exhaled breath [101]. Therefore, there is a possibility that the target organs differ between oral administration and intravenous administration, as suggested by Xie et al. [42]. However, the exposure of white blood cells in the blood to H_2_ is common situation in both cases. Thus, taken together the facts that organ-protective effects have been observed in animal experiments for ischemia/reperfusion injury [85] and in dialysis patients for the prevention of cardiovascular disease [104], it might be possible that we see H_2_ as having a secondary organ-protective effect that influences the reaction of macrophages rather than directly affecting organs [108,109]. Future investigations are needed to clarify this.

### 5.2. Amount of Added H_2_

H_2_ is a major byproduct produced by gut bacteria, and microbes able to utilize H_2_ as an electron donor by their hydrogenases [41]. In a study conducted in the 1960s on human subjects [110], it was reported that in healthy individuals, H_2_ production in the colon was around 15 mmol per day under normal conditions and reached over 100 mmol per day with lactose loading. It has been reported that 30–40% of the produced H_2_ is utilized by microbes, with the remaining H_2_ being excreted through breath or flatus [111]. On the other hand, in case of CKD model rats [21,84], the added amount of H_2_ in these experiments was about 0.1 mg/g body weight, which, when converted for a human weight of 50 kg, is roughly equivalent to adding approximately 1 mmol of H_2_. This level of H_2_ is equivalent to the amount of H_2_ load in clinical studies conducted on diabetes and metabolic syndrome. Despite the relatively low exogenous H_2_ load when considering the endogenous production of H_2_ in healthy individuals, the reason why H_2_ supplementation contributes to clinical effects remains unclear. Regarding the systemic impact, it is uncertain whether there are differences between exogenous and endogenous loads of H_2_ is a factor causing differences. Further investigations are needed to address these questions.

Nevertheless, considering the potential benefits of H_2_ supplementation, the clinical application of H_2_ should certainly be pursued. Water ionizers have evolved, and water ionizers capable of producing dissolved hydrogen concentrations up to 1300 ppm have been released since 2017. In the case of current electrolysis devices, the amount of EHW needed for H_2_ intake of 1 mmol varies depending on the model of the ionizer. We think that it is meaningful to conduct clinical studies in the future that clearly define CKD stage and underlying diseases.

Regarding HD, the potential increase in costs associated with using EHW is a crucial aspect that cannot be overlooked. In HD treatment, more than 150 L of water are used per session per person, and this applies similarly to HD using EHW. During the process of water electrolysis, the water generated on the cathode side is used, while the water on the anode side is discarded. The discarded water accounts for about 10% of the total treated water volume. Consequently, in dialysis systems using EHW, the required water volume is higher compared to conventional HD systems, resulting in increased treatment costs. In Japan, reimbursement for dialysis treatment is fixed (approximately 5–6 million yen per person per year), and since patients are fully exempt from the financial burden, the rise in treatment costs becomes a burden for medical institutions. In the future, considerations such as improving equipment performance and reusing discarded water need to be explored.

In conclusion, we discussed the potential antioxidant effects of EHW, attributing them to the physiological actions of H_2_. Although the antioxidant mechanisms of H_2_ are complex and still not fully understood, it is assumed that the activation of Nrf2 plays a central role. The progression of CKD, characterized by a decline in renal function due to kidney damage, is associated with increased oxidative stress within the kidneys. Additionally, as renal function decreases, systemic oxidative stress is enhanced. In HD patients, oxidative stress is further intensified during dialysis treatment due to the bio-incompatibility of dialysis equipment. Those state of increased oxidative stress serves as a risk factor for cardiovascular disease and susceptibility to infections. Therefore, the establishment of a long-term and safe antioxidant therapy is anticipated for patients with CKD and those undergoing HD. In this context, we believe that the medical application of EHW, already widely used in Japan, could be a significant candidate for this purpose. Whether drinking EHW can be a safe prophylaxis for CKD is a noteworthy challenge from the perspective of national health.

## Figures and Tables

**Table 1:** Effect of H_2_ water ad libitum drinking for CKD model rats.

CKD Model	H_2_ Load	Intervention	Observation	Main Findings	Oxidative Stress Marker
Dahl salt-sensitive rat (4 weeks old) [21]	Ad libitum drinking in respective groups (*n* = 30 each) EHW (H_2_: 0.49 mg/L) DW (H2: 0.003 mg/L) FW (H2: <0.001 mg/L)	N/A (fed by low sodium diet)	48 weeks	No striking differences in BP among 3 groups. Lower in EHW than DW and FW in cardiac remodeling, glomerular sclerosis with tubulointerstitial fibrosis in the kidney, and increased cardiomyocyte diameter with interstitial fibrosis in the heart.	Kidney: fewer nitrotyrosine, malondialdehyde, and ED1 cells in EHW than FW. Heart: less malondialdehyde in EW than FW, significantly higher Nrf2, and lower NADPH oxidase expression in EHW than FW.
Spontaneous hypertensive rat (8 weeks old) [84]	Ad libitum drinking in respective groups (*n* = 72 each) HW (H_2_: 0.8–1.3 mg/L) vehicle	N/A	12 weeks	No striking differences in BP between two groups. Lower glomerular sclerosis score and higher renal blood flow and glomerular filtration rate in HW than vehicle.	Lowered reactive oxygen species formation; upregulated the activities of superoxide dismutase, glutathione peroxidase, glutathione-S-epoxide transferase, and catalase, and suppressed NADPH oxidase in HW. Depressed pro-inflammatory cytokine expression in HW. Protective effect on mitochondrial function in HW.
Dahl salt-sensitive rat (7 weeks old) [85]	Ad libitum drinking in respective groups (*n* = 18 in EW, 17 in FW) EHW (H_2_: 0.35 mg/L) FW (H_2_: 0.0 mg/L)	Unilateral kidney I/R (fed a low sodium diet)	6 weeks of preconditioning followed by intervention and 1 week post observation	Contralateral kidney and heart: less glomerular adhesion, cardiac fibrosis in EHW.	Lower mRNA expression of NADPH oxidase 4 in heart in HW. Smaller number of ED-1 positive cells and nitrotyrosine in kidney and heart in EHW.

Abbreviations: CKD, chronic kidney disease; EHW, electrolyzed hydrogen water; DW, degassed electrolyzed hydrogen water; FW, filtered water; HW, hydrogen water; I/R, ischemia/reperfusion.

**Table 2:** Clinical trials of chronic HD employing electrolyzed hydrogen water.

Year (Ref.)	Study Design	H_2_ Level of HD Solution	Number of Patients	Duration	Outcome
2009 [100]	Single-arm	~99.0 ppb	8	1 month	Significant decrease of methylguanidine
2010 [101]	Single-arm	49 ppb (average)	21	6 months	BP reduction before and after HD Decrease of plasma MCP-1 and MPO (3rd tertile group)
2016 [102]	Parallel-arm	47–196 ppb	12 in EHD 38 in CHD	7 months	Significant elevation in serum reduced albumin fraction pre- and post-HD in EW-HD No differences between EHD (post) and healthy subjects
2017 [103]	Parallel-arm	30–80 ppb	140 in EHD 122 in CHD	12 months	Reduction of anti-hypertensive agents and subjective symptoms such as fatigue and pruritus
2018 [104]	Parallel-arm	30–80 ppb	161 in EHD 148 in CHD	3.28 years (average)	Reduction of post-HD BP in EHD Hazard ratio of EHD 0.59 for composite of all-cause mortality and non-lethal cardio-cerebrovascular events after adjusting for confounding factors
2021 [30]	Single-arm	41–81 ppb	63	2 months	Elevations of plasma MPO and thioredoxin at post-HD, elevation of plasma malondialdehyde at pre-HD and decrease at post-HD Decrease of VAS of fatigue
2021 [105]	Single-arm	120–163 ppb	95	2 months	Decrease of VAS of fatigue
2022 [106]	Single-arm	Basal 47 ppb (average) to 154 ppb (average) ppb	105	2 months	Decrease of plasma MPO pre-HD Decrease of NRS of fatigue

Abbreviations: BP, blood pressure; HD, hemodialysis; MCP-1, monocyte chemotactic protein 1; MPO, myeloperoxidase; EHD, hemodialysis employing electrolyzed hydrogen water; CHD, conventional hemodialysis; VAS, visual analogue scale; NRS, numerical rating scale.

## References

[1] Itokawa Y. (2004). An overview on researches of portable alkaline water by electrolysis. Kinousuikenkyu.

[2] Alkaline Ionized Water Apparatus Market Trends. https://www.3aaa.gr.jp/english/markettrend.html.

[3] Shirahata S., Kabayama S., Nakano M., Miura T., Kusumoto K., Gotoh M., Hayashi H., Otsubo K., Morisawa S., Katakura Y. (1997). Electrolyzed-reduced water scavenges active oxygen species and protects DNA from oxidative damage. Biochem. Biophys. Res. Commun..

[4] Li Y., Hamasaki T., Nakamichi N., Kashiwagi T., Komatsu T., Ye J., Teruya K., Abe M., Yan H., Kinjo T. (2011). Suppressive effects of electrolyzed reduced water on alloxan-induced apoptosis and type 1 diabetes mellitus. Cytotechnology.

[5] Yan H., Kinjo T., Tian H., Hamasaki T., Teruya K., Kabayama S., Shirahata S. (2011). Mechanism of the lifespan extension of Caenorhabditis elegans by electrolyzed reduced water—participation of Pt nanoparticles. Biosci. Biotechnol. Biochem..

[6] Wang Y., Fugetsu B., Sakata I., Fujisue C., Kabayama S., Tahara N., Morisawa S. (2020). Monolayered Platinum Nanoparticles as Efficient Electrocatalysts for the Mass Production of Electrolyzed Hydrogen Water. Sci. Rep..

[7] Ohsawa I., Ishikawa M., Takahashi K., Watanabe M., Nishimaki K., Yamagata K., Katsura K.-I., Katayama Y., Asoh S., Ohta S. (2007). Hydrogen acts as a therapeutic antioxidant by selectively reducing cytotoxic oxygen radicals. Nat. Med..

[8] Ichihara M., Sobue S., Ito M., Ito M., Hirayama M., Ohno K. (2015). Beneficial biological effects and the underlying mechanisms of molecular hydrogen—comprehensive review of 321 original articles. Med. Gas. Res..

[9] LeBaron T.W., Sharpe R., Ohno K. (2022). Electrolyzed-Reduced Water: Review I. Molecular Hydrogen Is the Exclusive Agent Responsible for the Therapeutic Effects. Int. J. Mol. Sci..

[10] Johnsen H.M., Hiorth M., Klaveness J. (2023). Molecular Hydrogen Therapy-A Review on Clinical Studies and Outcomes. Molecules.

[11] Mizuno K., Watanabe K., Yamano E., Ebisu K., Tajima K., Nojima J., Ohsaki Y., Kabayama S., Watanabe Y. (2022). Antioxidant effects of continuous intake of electrolyzed hydrogen water in healthy adults. Heliyon.

[12] Ohta S. (2014). Molecular hydrogen as a preventive and therapeutic medical gas: Initiation, development and potential of hydrogen medicine. Pharmacol. Ther..

[13] Zhang Y., Tan S., Xu J., Wang T. (2018). Hydrogen Therapy in Cardiovascular and Metabolic Diseases: From Bench to Bedside. Cell Physiol. Biochem..

[14] LeBaron T.W., Kura B., Kalocayova B., Tribulova N., Slezak J. (2019). A New Approach for the Prevention and Treatment of Cardiovascular Disorders. Molecular Hydrogen Significantly Reduces the Effects of Oxidative Stress. Molecules.

[15] Yang M., Dong Y., He Q., Zhu P., Zhuang Q., Shen J., Zhang X., Zhao M. (2020). Hydrogen: A Novel Option in Human Disease Treatment. Oxid. Med. Cell Longev..

[16] Tian Y., Zhang Y., Wang Y., Chen Y., Fan W., Zhou J., Qiao J., Wei Y. (2021). Hydrogen, a Novel Therapeutic Molecule, Regulates Oxidative Stress, Inflammation, and Apoptosis. Front. Physiol..

[17] Rahman M.H., Jeong E.-S., You H.S., Kim C.-S., Lee K.-J. (2023). Redox-Mechanisms of Molecular Hydrogen Promote Healthful Longevity. Antioxidants.

[18] Saengsin K., Sittiwangkul R., Chattipakorn S.C., Chattipakorn N. (2023). Hydrogen therapy as a potential therapeutic intervention in heart disease: From the past evidence to future application. Cell Mol. Life Sci..

[19] Suzuki T., Yamamoto M. (2017). Stress-sensing mechanisms and the physiological roles of the Keap1-Nrf2 system during cellular stress. J. Biol. Chem..

[20] Suzuki T., Takahashi J., Yamamoto M. (2023). Molecular Basis of the KEAP1-NRF2 Signaling Pathway. Mol. Cells..

[21] Zhu W.J., Nakayama M., Mori T., Hao K., Terawaki H., Katoh J., Kabayama S., Ito S. (2013). Amelioration of cardio-renal injury with aging in dahl salt-sensitive rats by H2-enriched electrolyzed water. Med. Gas. Res..

[22] Yuan J., Wang D., Liu Y., Chen X., Zhang H., Shen F., Liu X., Fu J. (2018). Hydrogen-rich water attenuates oxidative stress in rats with traumatic brain injury via Nrf2 pathway. J. Surg. Res..

[23] Kura B., Bagchi A.K., Singal P.K., Barancik M., LeBaron T.W., Valachova K., Šoltés L., Slezák J. (2019). Molecular hydrogen: Potential in mitigating oxidative-stress-induced radiation injury. Can. J. Physiol. Pharmacol..

[24] Yu Y., Feng J., Lian N., Yang M., Xie K., Wang G., Wang C., Yu Y. (2020). Hydrogen gas alleviates blood-brain barrier impairment and cognitive dysfunction of septic mice in an Nrf2-dependent pathway. Int. Immunopharmacol..

[25] Lu Y., Li C.F., Ping N.N., Sun Y.Y., Wang Z., Zhao G.X., Yuan S.H., Zibrila A.I., Soong L., Liu J.J. (2020). Hydrogen-rich water alleviates cyclosporine A-induced nephrotoxicity via the Keap1/Nrf2 signaling pathway. J. Biochem. Mol. Toxicol..

[26] Hu Y., Wang P., Han K. (2022). Hydrogen Attenuated Inflammation Response and Oxidative in Hypoxic Ischemic Encephalopathy via Nrf2 Mediated the Inhibition of NLRP3 and NF-κB. Neuroscience.

[27] Peng J., He Q., Li S., Liu T., Zhang J. (2022). Hydrogen-Rich Water Mitigates LPS-Induced Chronic Intestinal Inflammatory Response in Rats via Nrf-2 and NF-κB Signaling Pathways. Vet. Sci..

[28] Murakami Y., Ito M., Ohsawa I. (2017). Molecular hydrogen protects against oxidative stress-induced SH-SY5Y neuroblastoma cell death through the process of mitohormesis. PLoS ONE.

[29] Hirayama M., Ito M., Minato T., Yoritaka A., LeBaron T.W., Ohno K. (2019). Inhalation of hydrogen gas elevates urinary 8-hydroxy-2’-deoxyguanine in Parkinson’s disease. Med. Gas. Res..

[30] Satta H., Iwamoto T., Kawai Y., Koguchi N., Shibata K., Kobayashi N., Yoshida M., Nakayama M. (2021). Amelioration of hemodialysis-induced oxidative stress and fatigue with a hemodialysis system employing electrolyzed water containing molecular hydrogen. Ren. Replace. Ther..

[31] LeBaron T.W., Laher I., Kura B., Slezak J. (2019). Hydrogen gas: From clinical medicine to an emerging ergogenic molecule for sports athletes 1. Can J. Physiol. Pharmacol..

[32] Satoh T., McKercher S.R., Lipton S.A. (2013). Nrf2/ARE-mediated antioxidant actions of pro-electrophilic drugs. Free Radic. Biol. Med..

[33] Satoh T., Lipton S.A. (2007). Redox regulation of neuronal survival mediated by electrophilic compounds. Trends Neurosci..

[34] Calabrese V., Cornelius C., Dinkova-Kostova A.T., Calabrese E.J., Mattson M.P. (2010). Cellular stress responses, the hormesis paradigm, and vitagenes: Novel targets for therapeutic intervention in neurodegenerative disorders. Antioxid. Redox Signal..

[35] Shingu C., Koga H., Hagiwara S., Matsumoto S., Goto K., Yokoi I., Noguchi T. (2010). Hydrogen-rich saline solution attenuates renal ischemia-reperfusion injury. J. Anesth..

[36] Gvozdjáková A., Kucharská J., Kura B., Vančová O., Rausová Z., Sumbalová Z., Uličná O., Slezák J. (2020). A new insight into the molecular hydrogen effect on coenzyme Q and mitochondrial function of rats. Can. J. Physiol. Pharmacol..

[37] Huang Y., Xin W., Xiong J., Yao M., Zhang B., Zhao J. (2022). The Intestinal Microbiota and Metabolites in the Gut-Kidney-Heart Axis of Chronic Kidney Disease. Front. Pharmacol..

[38] Han B., Sivaramakrishnan P., Lin C.J., Neve I.A.A., He J., Tay L.W.R., Sowa J.N., Sizovs A., Du G., Wang J. (2017). Microbial Genetic Composition Tunes Host Longevity. Cell.

[39] Matsuhashi T., Sato T., Kanno S.I., Suzuki T., Matsuo A., Oba Y., Kikusato M., Ogasawara E., Kudo T., Suzuki K. (2017). Mitochonic Acid 5 (MA-5) Facilitates ATP Synthase Oligomerization and Cell Survival in Various Mitochondrial Diseases. EBioMedicine.

[40] Yardeni T., Tanes C.E., Bittinger K., Mattei L.M., Schaefer P.M., Singh L.N., Wu G.D., Murdock D.G., Wallace D.C. (2019). Host mitochondria influence gut microbiome diversity: A role for ROS. Sci. Signal..

[41] Wolf P.G., Biswas A., Morales S.E., Greening C., Gaskins H.R. (2016). H2 metabolism is widespread and diverse among human colonic microbes. Gut Microbes.

[42] Xie F., Jiang X., Yi Y., Liu Z.J., Ma C., He J., Xun Z.M., Wang M., Liu M.Y., Mawulikplimi Adzavon Y. (2022). Different effects of hydrogen-rich water intake and hydrogen gas inhalation on gut microbiome and plasma metabolites of rats in health status. Sci. Rep..

[43] Matsushita K., Ballew S.H., Wang A.Y., Kalyesubula R., Schaeffner E., Agarwal R. (2022). Epidemiology and risk of cardiovascular disease in populations with chronic kidney disease. Nat. Rev. Nephrol..

[44] Leung K.C., Tonelli M., James M.T. (2013). Chronic kidney disease following acute kidney injury-risk and outcomes. Nat. Rev. Nephrol..

[45] Kishi S., Nagasu H., Kidokoro K., Kashihara N. (2023). Oxidative stress and the role of redox signalling in chronic kidney disease. Nat. Rev. Nephrol..

[46] Verma S., Singh P., Khurana S., Ganguly N.K., Kukreti R., Saso L., Rana D.S., Taneja V., Bhargava V. (2021). Implications of oxidative stress in chronic kidney disease: A review on current concepts and therapies. Kidney Res. Clin. Pract..

[47] Himmelfarb J., Stenvinkel P., Ikizler T.A., Hakim R.M. (2002). The elephant in uremia: Oxidant stress as a unifying concept of cardiovascular disease in uremia. Kidney Int..

[48] Stenvinkel P., Chertow G.M., Devarajan P., Levin A., Andreoli S.P., Bangalore S., Warady B.A. (2021). Chronic Inflammation in Chronic Kidney Disease Progression: Role of Nrf2. Kidney Int. Rep..

[49] Manning R.D., Tian N., Meng S. (2005). Oxidative stress and antioxidant treatment in hypertension and the associated renal damage. Am. J. Nephrol..

[50] Hong Y.A., Park C.W. (2021). Catalytic Antioxidants in the Kidney. Antioxidants.

[51] Lo R., Narasaki Y., Lei S., Rhee C.M. (2023). Management of traditional risk factors for the development and progression of chronic kidney disease. Clin. Kidney J..

[52] Brand S., Amann K., Mandel P., Zimnol A., Schupp N. (2014). Oxidative DNA damage in kidneys and heart of hypertensive mice is prevented by blocking angiotensin II and aldosterone receptors. PLoS ONE.

[53] Li J., Liu H., Takagi S., Nitta K., Kitada M., Srivastava S.P., Takagaki Y., Kanasaki K., Koya D. (2020). Renal protective effects of empagliflozin via inhibition of EMT and aberrant glycolysis in proximal tubules. JCI Insight..

[54] de Zeeuw D., Akizawa T., Audhya P., Bakris G.L., Chin M., Christ-Schmidt H., Goldsberry A., Houser M., Krauth M., Lambers Heerspink H.J. (2013). BEACON Trial Investigators. Bardoxolone methyl in type 2 diabetes and stage 4 chronic kidney disease. N. Engl. J. Med..

[55] Bolati D., Shimizu H., Yisireyili M., Nishijima F., Niwa T. (2013). Indoxyl sulfate, a uremic toxin, downregulates renal expression of Nrf2 through activation of NF-κB. BMC Nephrol..

[56] Watanabe H., Miyamoto Y., Honda D., Tanaka H., Wu Q., Endo M., Noguchi T., Kadowaki D., Ishima Y., Kotani S. (2013). p-Cresyl sulfate causes renal tubular cell damage by inducing oxidative stress by activation of NADPH oxidase. Kidney Int..

[57] Miyazawa N., Abe M., Souma T., Tanemoto M., Abe T., Nakayama M., Ito S. (2010). Methylglyoxal augments intracellular oxidative stress in human aortic endothelial cells. Free Radic. Res..

[58] Richter B., Haller J., Haffner D., Leifheit-Nestler M. (2016). Klotho modulates FGF23-mediated NO synthesis and oxidative stress in human coronary artery endothelial cells. Pflugers Arch..

[59] Bowry S.K., Kircelli F., Himmele R., Nigwekar S.U. (2021). Blood-incompatibility in haemodialysis: Alleviating inflammation and effects of coagulation. Clin. Kidney J..

[60] Zuo J., Chaykovska L., Chu C., Chen X., Hasan A.A., Krämer B.K., Tepel M., Hocher B. (2022). Head-to-Head Comparison of Oxidative Stress Biomarkers for All-Cause Mortality in Hemodialysis Patients. Antioxidants.

[61] Colombijn J.M., Hooft L., Jun M., Webster A.C., Bots M.L., Verhaar M.C., Vernooij R.W. (2023). Antioxidants for adults with chronic kidney disease. Cochrane Database Syst. Rev..

[62] Wang B., Li Z., Mao L., Zhao M., Yang B., Tao X., Li Y., Yin G. (2022). Hydrogen: A Novel Treatment Strategy in Kidney Disease. Kidney Dis..

[63] Hirano S.I., Ichikawa Y., Sato B., Takefuji Y., Satoh F. (2023). Clinical Use and Treatment Mechanism of Molecular Hydrogen in the Treatment of Various Kidney Diseases including Diabetic Kidney Disease. Biomedicines..

[64] Yao W., Guo A., Han X., Wu S., Chen C., Luo C., Li H., Li S., Hei Z. (2019). Aerosol inhalation of a hydrogen-rich solution restored septic renal function. Aging.

[65] Kawamura M., Imamura R., Kobayashi Y., Taniguchi A., Nakazawa S., Kato T., Namba-Hamano T., Abe T., Uemura M., Kobayashi H. (2020). Oral administration of Si-based agent attenuates oxidative stress and ischemia-reperfusion injury in a rat model: A novel hydrogen administration method. Front. Med..

[66] Li J., Hong Z., Liu H., Zhou J., Cui L., Yuan S., Chu X., Yu P. (2016). Hydrogen-rich saline promotes the recovery of renal function after ischemia/reperfusion injury in rats via anti-apoptosis and anti-inflammation. Front. Pharmacol..

[67] Xu X., He X., Liu J., Qin J., Ye J., Fan M. (2019). Protective effects of hydrogen-rich saline against renal ischemia-reperfusion injury by increased expression of heme oxygenase-1 in aged rats. Int. J. Clin. Exp. Pathol..

[68] Nishida T., Hayashi T., Inamoto T., Kato R., Ibuki N., Takahara K., Yoshikawa Y., Uchimoto T., Saito K., Tanda N. (2018). Dual gas treatment with hydrogen and carbon monoxide attenuates oxidative stress and protects from renal ischemia-reperfusion injury. Transplant. Proc..

[69] Du H., Sheng M., Wu L., Zhang Y., Shi D., Weng Y., Xu R., Yu W. (2016). Hydrogen-rich saline attenuates acute kidney injury after liver transplantation via activating p53-mediated autophagy. Transplantation..

[70] Wang F., Yu G., Liu S.Y., Li J.B., Wang J.F., Bo L.L., Qian L.R., Sun X.J., Deng X.M. (2011). Hydrogen-rich saline protects against renal ischemia/reperfusion injury in rats. J. Surg. Res..

[71] Chen J., Zhang H., Hu J., Gu Y., Shen Z., Xu L., Jia X., Zhang X., Ding X. (2017). Hydrogen-rich saline alleviates kidney fibrosis following AKI and retains Klotho expression. Front. Pharmacol..

[72] Liu W., Dong X.S., Sun Y.Q., Liu Z. (2014). A novel fluid resuscitation protocol: Provide more protection on acute kidney injury during septic shock in rats. Int. J. Clin. Exp. Med..

[73] Shi Q., Liao K.S., Zhao K.L., Wang W.X., Zuo T., Deng W.H., Chen C., Yu J., Guo W.Y., He X.B. (2015). Hydrogen-rich saline attenuates acute renal injury in sodium taurocholate-induced severe acute pancreatitis by inhibiting ROS and NF-κB pathway. Mediators Inflamm..

[74] Guan P., Sun Z.M., Luo L.F., Zhou J., Yang S., Zhao Y.S., Yu F.Y., An J.R., Wang N., Ji E.S. (2019). Hydrogen protects against chronic intermittent hypoxia induced renal dysfunction by promoting autophagy and alleviating apoptosis. Life Sci..

[75] Cardinal J.S., Zhan J., Wang Y., Sugimoto R., Tsung A., McCurry K.R., Billiar T.R., Nakao A. (2010). Oral hydrogen water prevents chronic allograft nephropathy in rats. Kidney Int..

[76] Abe T., Li X.K., Yazawa K., Hatayama N., Xie L., Sato B., Kakuta Y., Tsutahara K., Okumi M., Tsuda H. (2012). Hydrogen-rich University of Wisconsin solution attenuates renal cold ischemia-reperfusion injury. Transplantation.

[77] Nakashima-Kamimura N., Mori T., Ohsawa I., Asoh S., Ohta S. (2009). Molecular hydrogen alleviates nephrotoxicity induced by an anti-cancer drug cisplatin without compromising anti-tumor activity in mice. Cancer Chemother. Pharmacol..

[78] Li F.Y., Zhu S.X., Wang Z.P., Wang H., Zhao Y., Chen G.P. (2013). Consumption of hydrogen-rich water protects against ferric nitrilotriacetate-induced nephrotoxicity and early tumor promotional events in rats. Food Chem. Toxicol..

[79] Peng Z., Chen W., Wang L., Ye Z., Gao S., Sun X., Guo Z. (2015). Inhalation of hydrogen gas ameliorates glyoxylate-induced calcium oxalate deposition and renal oxidative stress in mice. Int. J. Clin. Exp. Pathol..

[80] Lu H., Ding J., Liu W., Peng Z., Chen W., Sun X., Guo Z. (2018). UPLC/MS-based metabolomics investigation of the protective effect of hydrogen gas inhalation on mice with calcium oxalate-induced renal injury. Biol. Pharm. Bull..

[81] Xu B., Zhang Y.B., Li Z.Z., Yang M.W., Wang S., Jiang D.P. (2013). Hydrogen-rich saline ameliorates renal injury induced by unilateral ureteral obstruction in rats. Int. Immunopharmacol..

[82] Xing Z., Pan W., Zhang J., Xu X., Zhang X., He X., Fan M. (2017). Hydrogen rich water attenuates renal injury and fibrosis by regulation transforming growth factor-β induced Sirt1. Biol. Pharm. Bull..

[83] Mizutani A., Endo A.A., Saito M., Hara T., Nakagawa M., Sakuraya K., Murano Y., Nishizaki N., Hirano D., Fujinaga S. (2022). Hydrogen-rich water reduced oxidative stress and renal fibrosis in rats with unilateral ureteral obstruction. Pediatr. Res..

[84] Xin H.G., Zhang B.B., Wu Z.Q., Hang X.F., Xu W.S., Ni W., Zhang R.Q., Miao X.H. (2014). Consumption of hydrogen-rich water alleviates renal injury in spontaneous hypertensive rats. Mol. Cell Biochem..

[85] Zhu W.J., Nakayama M., Mori T., Nakayama K., Katoh J., Murata Y., Sato T., Kabayama S., Ito S. (2011). Intake of water with high levels of dissolved hydrogen (H_2_) suppresses ischemia-induced cardio-renal injury in Dahl salt-sensitive rats. Nephrol. Dial. Transplant..

[86] Kelly K.J. (2003). Distant effects of experimental renal ischemia/reperfusion injury. J. Am. Soc. Nephrol..

[87] Sugai K., Tamura T., Sano M., Uemura S., Fujisawa M., Katsumata Y., Endo J., Yoshizawa J., Homma K., Suzuki M. (2020). Daily inhalation of hydrogen gas has a blood pressure-lowering effect in a rat model of hypertension. Sci. Rep..

[88] Inoue T. (2021). Neuroimmune system-mediated renal protection mechanisms. Clin. Exp. Nephrol..

[89] Xie F., Song Y., Yi Y., Jiang X., Ma S., Ma C., Li J., Zhanghuang Z., Liu M., Zhao P. (2023). Therapeutic Potential of Molecular Hydrogen in Metabolic Diseases from Bench to Bedside. Pharmaceuticals.

[90] Kajiyama S., Hasegawa G., Asano M., Hosoda H., Fukui M., Nakamura N., Kitawaki J., Imai S., Nakano K., Ohta M. (2008). Supplementation of hydrogen-rich water improves lipid and glucose metabolism in patients with type 2 diabetes or impaired glucose tolerance. Nutr. Res..

[91] Ogawa S., Ohsaki Y., Shimizu M., Nako K., Okamura M., Kabayama S., Tabata K., Tanaka Y., Ito S. (2021). Electrolyzed hydrogen-rich water for oxidative stress suppression and improvement of insulin resistance: A multicenter prospective double-blind randomized control trial. Diabetol. Int..

[92] Nakao A., Toyoda Y., Sharma P., Evans M., Guthrie N. (2010). Effectiveness of hydrogen rich water on antioxidant status of subjects with potential metabolic syndrome-an open label pilot study. J. Clin. Biochem. Nutr..

[93] Liu B., Jiang X., Xie Y., Jia X., Zhang J., Xue Y., Qin S. (2022). The effect of a low dose hydrogen-oxygen mixture inhalation in midlife/older adults with hypertension: A randomized, placebo-controlled trial. Front. Pharmacol..

[94] Sumida K., Yamagata K., Kovesdy C.P. (2020). Constipation in CKD. Kidney Int. Rep..

[95] Cosola C., Rocchetti M.T., di Bari I., Acquaviva P.M., Maranzano V., Corciulo S., Di Ciaula A., Di Palo D.M., La Forgia F.M., Fontana S. (2021). An Innovative Synbiotic Formulation Decreases Free Serum Indoxyl Sulfate, Small Intestine Permeability and Ameliorates Gastrointestinal Symptoms in a Randomized Pilot Trial in Stage IIIb-IV CKD Patients. Toxins.

[96] Huang K.C., Yang C.C., Lee K.T., Chien C.T. (2003). Reduced hemodialysis-induced oxidative stress in end-stage renal disease patients by electrolyzed reduced water. Kidney Int..

[97] Huang K.C., Yang C.C., Hsu S.P., Lee K.T., Liu H.W., Morisawa S., Otsubo K., Chien C.T. (2006). Electrolyzed-reduced water reduced hemodialysis-induced erythrocyte impairment in end-stage renal disease patients. Kidney Int..

[98] Tange Y., Takesawa S., Yoshitake S. (2015). Dialysate with high dissolved hydrogen facilitates dissociation of indoxyl sulfate from albumin. Nephrourol. Mon..

[99] Nakayama M., Kabayama S., Ito S. (2016). The hydrogen molecule as antioxidant therapy: Clinical application in hemodialysis and perspectives. Ren. Replace. Ther..

[100] Nakayama M., Kabayama S., Nakano H., Zhu W.J., Terawaki H., Nakayama K., Katoh K., Satoh T., Ito S. (2009). Biological effects of electrolyzed water in hemodialysis. Nephron Clin. Pract..

[101] Nakayama M., Nakano H., Hamada H., Itami N., Nakazawa R., Ito S. (2010). A novel bioactive haemodialysis system using dissolved dihydrogen (H2) produced by water electrolysis: A clinical trial. Nephrol. Dial. Transplant..

[102] Maeda K., Yoshizaki S., Iida T., Terada T., Era S., Sakashita K., Arikawa H. (2016). Improvement of the fraction of human mercaptalbumin on hemodialysis treatment using hydrogen-dissolved hemodialysis fluid: A prospective observational study. Ren. Replace. Ther..

[103] Nakayama M., Itami N., Suzuki H., Hamada H., Osaka N., Yamamoto R., Tsunoda K., Nakano H., Watanabe K., Zhu W.J. (2017). in a 12 month observation. PLoS ONE.

[104] Nakayama M., Itami N., Suzuki H., Hamada H., Yamamoto R., Tsunoda K., Osaka N., Nakano H., Maruyama Y., Kabayama S. (2018). Novel haemodialysis (HD) treatment employing molecular hydrogen (H_2_)-enriched dialysis solution improves prognosis of chronic dialysis patients: A prospective observational study. Sci. Rep..

[105] Tsujimoto Y., Kuratsune D., Kabayama S., Miyazaki M., Watanabe Y., Nishizawa Y., Nakayama M. (2021). Amelioration of fatigue in chronic dialysis patients with dialysis solution employing electrolyzed water containing molecular hydrogen (H_2_) and its association with autonomic function balance. Ren. Replace. Ther..

[106] Uemura S., Kegasa Y., Tada K., Tsukahara T., Kabayama S., Yamamoto T., Miyazaki M., Takada J., Nakayama M. (2022). Impact of hemodialysis solutions containing different levels of molecular hydrogen (H_2_) on the patient-reported outcome of fatigue. Ren. Replace. Ther..

[107] Mouzakis F.L., Khadka L.B., da Silva M.P., Mottaghy K. (2022). Quantification of dissolved H_2_ and continuous monitoring of hydrogen-rich water for haemodialysis applications: An experimental study. Int. J. Artif. Organs.

[108] Pérez S., Rius-Pérez S. (2022). Macrophage Polarization and Reprogramming in Acute Inflammation: A Redox Perspective. Antioxidants.

[109] Liu P., Liu D., Chen F., Luo L., Jin Y., Peng J., Yu H., Wei M., Shi X., Wang L. (2022). Effect of Nrf2 on Phenotype Changes of Macrophages in the Anterior Vaginal Wall of Women with Pelvic Organ Prolapse. Urogynecology.

[110] Levitt M.D. (1969). Production and excretion of hydrogen gas in man. N. Engl. J. Med..

[111] Christl S.U., Murgatroyd P.R., Gibson G.R., Cummings J.H. (1992). Production, metabolism, and excretion of hydrogen in the large intestine. Gastroenterology.

